# Emotional dysregulation, alexithymia and neuroticism: a systematic review on the genetic basis of a subset of psychological traits

**DOI:** 10.1097/YPG.0000000000000335

**Published:** 2022-12-20

**Authors:** Giovanni Castellini, Giuseppe Pierpaolo Merola, Ottone Baccaredda Boy, Vincenzo Pecoraro, Bernardo Bozza, Emanuele Cassioli, Eleonora Rossi, Valentina Bessi, Sandro Sorbi, Benedetta Nacmias, Valdo Ricca

**Affiliations:** a Psychiatry Unit; b Neurology Unit, Department of Health Sciences, University of Florence, Florence, Italy

**Keywords:** alexithymia, emotion dysregulation, genetic, genome-wide association study, neuroticism

## Abstract

Neuroticism, alexithymia and emotion dysregulation are key traits and known risk factors for several psychiatric conditions. In this systematic review, the aim is to evaluate the genetic contribution to these psychological phenotypes. A systematic review of articles found in PubMed was conducted. Search terms included ‘genetic’, ‘GWAS’, ‘neuroticism’, ‘alexithymia’ and ‘emotion dysregulation’. Risk of bias was assessed utilizing the STREGA checklist. Two hundred two papers were selected from existing literature based on the inclusion and exclusion criteria. Among these, 27 were genome-wide studies and 175 were genetic association studies. Single gene association studies focused on selected groups of genes, mostly involved in neurotransmission, with conflicting results. GWAS studies on neuroticism, on the other hand, found several relevant and replicated intergenic and intronic loci affecting the expression and regulation of crucial and well-known genes (such as DRD2 and CRHR1). Mutations in genes coding for trascriptional factors were also found to be associated with neuroticism (DCC, XKR6, TCF4, RBFOX1), as well as a noncoding regulatory RNA (LINC00461). On the other hand, little GWAS data are available on alexythima and emotional dysregulation.

## Introduction

It is now widely accepted that the psychological framework of each individual is shaped by a complex interplay of environmental, biological and genetic factors; the latter constitute an intriguing field of research, as a better understanding of the genomic variants concurring to a specific personality archetype or psychological trait may serve as an invaluable tool to comprehend and predict the intricacies of personal and collective mechanisms of information processing. Genetics has played a prominent role in psychiatric research since the very beginning of the era of etiological investigation of mental diseases (Burmeister *et al*., 2008), especially, regarding bipolar disorder and schizophrenia (Escamilla and Zavala, 2008; Henriksen *et al*., 2017). This intuition was later applied also to psychological and personality traits (Jang *et al*., 1996). Psychiatry genetics also benefitted greatly from recent advances in sequencing technologies; large-scale investigations such as genome-wide association studies (GWASs), in which thousands or even hundreds of thousands of genomes are correlated to a specific trait with a hypothesis-free approach, rather than considering a single gene-trait interconnection (Collins and Sullivan, 2013; Visscher *et al*., 2017), are now feasible.

Starting from this promising foothold, research into the field has expanded exponentially; among the various psychopathological domains, personality traits were one of the most obvious subjects to investigate, due to their unambiguous characterization (Rao and Broadbear, 2019), which retains its validity even in presence of potentially confounding sociocultural factors (Ayinde and Gureje, 2021) (at least, if compared to other psychological entities) (Zimmerman *et al*., 2015).

Most of the research on these topics employs the NEO Personality Inventory (named after its main original components, neuroticism, extraversion and openness to experiences) the same paper is already cited shortly after (McCrae), a well-recognized tool that explores five personality dimensions while providing an unparalleled perspective on multiple psychological domains (McCrae *et al*., 2005).

Some of these traits share numerous features and, rather than being distinct entities, contribute to complex personality structures that in certain occasions also predispose to psychiatric morbidity. One such case is constituted by alexithymia, emotional dysregulation and neuroticism, which all fall into a category characterized by inadequate, exaggerated or inhibited emotional responses, with frequent mood shifts and increased incidence of psychiatric conditions (Bradley *et al*., 2011; Ormel *et al*., 2013; Hemming *et al*., 2019). These entities in turn serve as a manifestation of this spectrum of features, hence identifying their common genetic roots might serve as an adequate instrument to describe the higher rank system encompassing them. These traits have an estimated heritability factor between 30 and 60% (evaluated through twin concordance studies) (Picardi *et al*., 2011; Hawn *et al*., 2015; Boomsma *et al*., 2018).

Several association analyses have been carried out on these traits (see the Materials and methods section); however, an investigation on their causative factors while considering them as a complex, multifacet – but still individual – entity might provide a whole new perspective on the subject. As shedding light on these constructs might prompt the development of new clinical and procedural approaches, and better understanding of their genetic basis might serve as a solid starting point, a review concerning these specific personality traits and their gene associations might as well prove valuable. To achieve this, the first step was to clearly define these traits separately, to subsequently merge the inferred information in a comprehensive report.

### Neuroticism

Neuroticism as a concept was first theorized explicitly by Eysenck around 1950 (Eysenck, 1952).

People high in neuroticism (both meant as test scores and theoretical conception) are more prone to negative emotions than average; they also tend to be more impulsive, less able to delay gratification and more likely to suffer from life stressors. Neuroticism can, thus, be defined as the tendency to experience negative emotions such as fear, anxiety, irritability, feelings of guilt and anger (Widiger and Oltmanns, 2007). These difficulties often heavily influence the individual’s ability to navigate effectively social contexts (Messina *et al*., 2010).

The concept of neuroticism is not only useful in the field of personality psychology but also when taking into account psychiatric clinical practice. In fact, it has been proven that people with high neuroticism scores have a higher chance of being diagnosed with a mental health disorder, especially regarding internalizing conditions such as anxiety and depressive disorders (Khan *et al*., 2005; Gale *et al*., 2016).

Currently, neuroticism is measured through a wide variety of scales. Perhaps the most well-known is the NEO Personality Inventory, a test made up of 240 items (McCrae *et al*., 2005). Other tests often used are Eysenck Personality Inventory (EPI) and Eysenck Personality Questionnaire (EPQ) (Knowles and Kreitman, 1965; Loo, 1979). Both these tests have a similar outlook on what ‘neuroticism’ means while differing in some aspects (Rocklin and Revelle, 1981).

### Alexithymia

The alexithymia construct, first introduced by Nemiah and Sifneos in the early 70s (Sifneos, 1986), was the point of arrival of a decades-long research into the cognitive style of patients with psychosomatic diseases. The salient features initially identified encompassed the difficulty in recognizing and describing feelings in the context of an externally oriented cognitive style, as well as the perplexity in distinguishing between feelings and the bodily sensations of emotional arousal. This inability to correctly interpret bodily inputs can often lead to psychosomatic symptoms and reduced insight. Moreover, alexithymia has been linked to psychopathology in the spheres of borderline personality disorder, eating disorders and psychosis (Mannarini *et al*., 2016). Neuroscientific studies on alexithymia point toward the importance of amygdala functioning (Goerlich, 2018).

The main tools for the evaluation of alexithymia are the Toronto Alexithymia Scale (Taylor *et al*., 2003) and the Schalling-Sifneos Personality Scale (Sifneos, 1986). Alexithymia has been often associated with several psychopathological conditions, most prominently depression and eating disorders (Taylor, 1984). Upon the realization of its validity in the clinical and research fields, alexithymia was included as an item in various assessment and diagnostic questionnaires and furtherly investigated by dedicated inventories (Sifneos, 1973; Montagne *et al*., 2007; de Vroege *et al*., 2018).

### Emotion dysregulation

Emotion dysregulation (ED) is a wide and multifacet psychological concept (Thompson, 2019). It encompasses several different psychological traits and is associated with Borderline personality disorder (Carpenter and Trull, 2013), autism spectrum disorders (Cai *et al*., 2018), attention deficit hyperactivity disorder (Shaw *et al*., 2014), post traumatic disorder (Powers *et al*., 2015) and bipolar disorder (Bayes *et al*., 2016). Moreover, it often leads to substance abuse (Garke *et al*., 2021), self-harm (Gratz and Roemer, 2008) or even suicidal behavior (Raudales *et al*., 2020). ED is thought to be connected to abuse and trauma, especially during childhood (Dvir *et al*., 2014). This personality feature is characterized by incapacity or difficulty in modulating emotions in order to fit them to the social context. People with ED often suffer from poorer attention, labile mood and overly intense emotions. This trait often produces abnormal behavior both in the externalizing and internalizing spectrum. ED has been shown to be linked to neuroticism (Paulus *et al*., 2016), and interestingly, there is even evidence of this connection from a neuroscientific standpoint (Yang *et al*., 2020; Silverman *et al.*, 2019). According to Gratz and Roemer (2004), ED can be defined as a deficit in awareness and acceptance of emotions, with a lack of control of one’s impulsive behavior. As such, ED often results in difficulties in employing appropriate strategies in social contexts.

Due to its broad definition, ED is investigated through a variety of scales, such as Difficulties in Emotion Regulation Scale (Gratz and Roemer, 2004), Emotion Dysregulation Scale (Powers *et al*., 2015), Emotional Expressivity Scale (Burgin *et al*., 2012), Connor-Davidson Resilience Scale (Connor and Davidson, 2003), Emotion Regulation Checklist (Shields and Cicchetti, 1997), Personality Assessment Inventory (Venta *et al*., 2018) and Sleep and Emotional Reactivity in Alcohol Use Disorder (NCT04979507, 2021). The multitude of instruments employed to assess ED might strengthen the belief that it might be a somewhat ill-defined concept in current literature (Cole, 2014); this translates into the tendency to study it from different perspectives and angles. However, such a tendency might as well negatively affect the reproducibility of the samples, which were attributed this trait by using different scales.

### Objective

The aim of this systematic review is to investigate the influence of genetics on these three traits and to categorize known data on the subject, both from a GWAS and genetic association study perspective.

## Materials and methods

This review adheres to the 2020 PRISMA guidelines (Page *et al*., 2021).

### Eligibility criteria

Included articles were observational studies, either cross-sectional or longitudinal. The inclusion criteria were as follows: original article, written in English, reporting results of genetic analysis on humans in combination with measurements of neuroticism, ED or alexithymia using validated tools (questionnaires or tasks). The main outcome measures were all associations of either neuroticism, ED or alexithymia with specific alleles or intergenic variants. Exclusion criteria were: the study being a systematic review, a meta-analysis, an opinion article and methodological or technical contributions with no analysis over clinical data.

### Information sources and search strategy

The authors used the electronic database PubMed in order to select studies. The following string was used for the systematic search:

(emotion regulation[Title/Abstract] OR emotion dysregulation[Title/Abstract] OR alexithymia[Title/Abstract] OR neuroticism[Title/Abstract]) AND (genetics OR gene OR genome OR GWAS OR genome-wide association study) NOT review[pt] NOT systematic review[pt] NOT meta-analysis[pt] AND eng[la].

The last search was run on 14 July 2021.

### Selection process

Three authors (O.B.B, G.P.M. and V.P.) independently assessed the abstracts of potentially eligible studies. Eligibility assessment was performed in an unblinded standardized manner. If there was doubt about whether the study was eligible for inclusion, the reviewers examined the full text of the articles. The published protocol required consensus in case the authors disagreed on the inclusion of a specific study. In case, the opinion was not unanimous, a majority vote would have been taken between all authors. The authors agreed on all the eligibility assessments of the studies, and no consensus vote needed to take place.

### Data collection process and data items

Four authors (O.B.B., V.P., G.P.M. and B.B.) independently extracted the following categories of data from each included study: study design (GWAS or genetic association), population (number of subjects, ethnicity), genes studied, polymorphisms and their effect on the traits.

### Risk of bias

Risk of bias for individual studies was assessed using the STrengtheningthe REportingof Genetic Association Studies variant of the STROBE checklist for genetic association studies (Little *et al*., 2009). GWAS were not assessed through risk of bias, as no proper tool is available for such aim. An online tool was used to produce the summary graph S1 (McGuinness and Higgins, 2021).

## Results

**Fig. 1:**
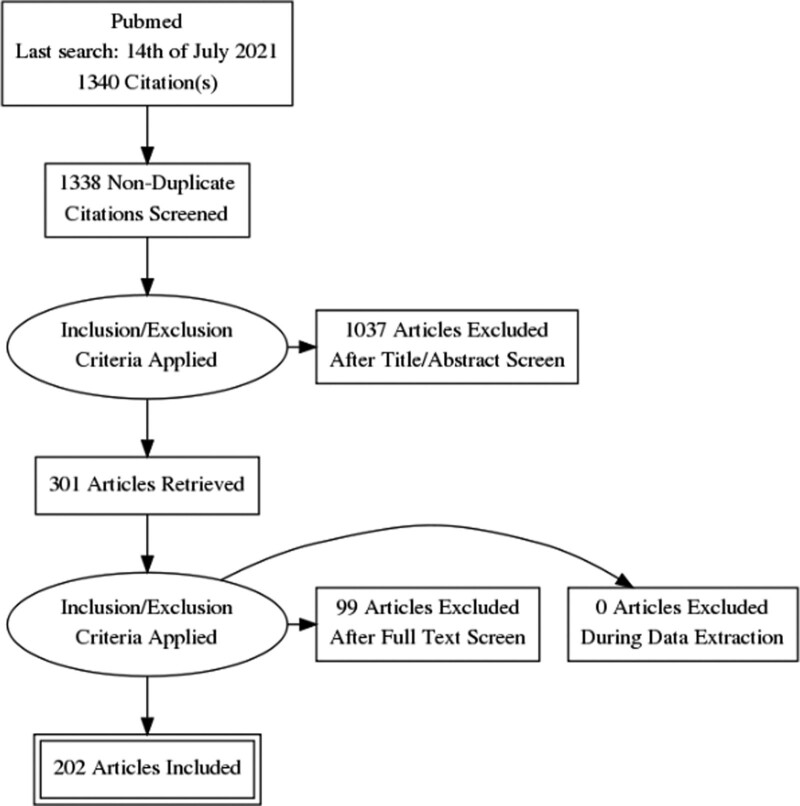
Flow diagram.

A total of 1340 studies were found after running the search line through PubMed. 301 studies were included for evaluation of the manuscript, 1039 were excluded on the basis of title and abstract and 99 were excluded after manuscript review and application of inclusion criteria. 202 studies (Rinieris *et al*., 1980; Ball *et al*., 1997; Gelernter *et al*., 1998; Jorm *et al*., 1998, 2000; Deary *et al*., 1999; Flory *et al*., 1999; Gustavsson *et al*., 1999; Sirota *et al*., 1999; Du *et al*., 2000; Greenberg *et al*., 2000; Henderson *et al*., 2000; Lerman *et al*., 2000; Persson *et al*., 2000; Sher *et al*., 2000; Jang *et al*., 2001; Close Kirkwood *et al*., 2002; Stoltenberg *et al*., 2002; Brummett *et al*., 2003; Eley *et al*., 2003; Fullerton *et al*., 2003; Sen *et al*., 2003, 2004; Umekage *et al*., 2003; Westberg *et al*., 2003, 2009; Jacob *et al*., 2004; Samochowiec *et al*., 2004; Tsai *et al*., 2004; Ham *et al*., 2005; Kato *et al*., 2005; Kusumi *et al*., 2005; Lang *et al*., 2005; Neale *et al*., 2005; Olsson *et al*., 2005; Tochigi *et al*., 2005, 2006; Wacker *et al*., 2005; Willis-Owen *et al*., 2005; Beem *et al*., 2006; Dragan and Oniszczenko, 2006; Hettema *et al*., 2006, 2008, 2009, 2013, 2015; Hibino *et al*., 2006; Hoth *et al*., 2006; Koller *et al*., 2006; van Rijn *et al*., 2006; Fiocco *et al*., 2007, 2009; Gatt *et al*., 2007; Gutknecht *et al*., 2007; Hünnerkopf *et al*., 2007; Middeldorp *et al*., 2007, 2010, 2010; Pascual *et al*., 2007; Urata *et al*., 2007; Waga and Iwahashi, 2007; Wasserman *et al*., 2007; Wray *et al*., 2007, 2008, 2008, 2009; Gillespie *et al*., 2008; Heck *et al*., 2008; Kazantseva *et al*., 2008, 2011; Rietschel *et al*., 2008; Shifman *et al*., 2008; van den Oord *et al*., 2008; Wachleski *et al*., 2008; Harro *et al*., 2009; Joffe *et al*., 2009; Juhasz *et al*., 2009, 2010; Kochanska *et al*., 2009; Murakami *et al*., 2009; Schmitz *et al*., 2009; Terracciano *et al*., 2009, 2010; Unschuld *et al*., 2009; Antypa and Van der Does, 2010; Calboli *et al*., 2010; Luciano *et al*., 2010, 2012, 2018, 2021; Vinberg *et al*., 2010; Zuo *et al*., 2010; DeYoung *et al*., 2011; Ellis *et al*., 2011; Swart *et al*., 2011; Verschoor and Markus, 2011; Walter *et al*., 2011; Amin *et al*., 2012; Amstadter *et al*., 2012; Boscarino *et al*., 2012; Dar-Nimrod *et al*., 2012; Jutras-Aswad *et al*., 2012; Kano *et al*., 2012; Kuepper *et al*., 2012; Kurrikoff *et al*., 2012; Markus and Capello, 2012; McIntosh *et al*., 2012; Montag *et al*., 2012, 2013; Pełka-Wysiecka *et al*., 2012; Propper *et al*., 2012; Wahlstrom *et al*., 2012; Wingbermühle *et al*., 2012; Aragam *et al*., 2013; Barbato *et al*., 2013; Glocke *et al*., 2013; Grazioplene *et al*., 2013; Kim *et al*., 2013, 2015; Kuhnen *et al*., 2013; Lehto *et al*., 2013, 2015, 2016; Mandelli *et al*., 2013; Markus, 2013; Strohmaier *et al*., 2013; Zayats *et al*., 2013; Chang *et al*., 2014, 2017, 2020; Criado *et al*., 2014; Gong *et al*., 2014; Haram *et al*., 2014; Laas *et al*., 2014; Lovallo *et al*., 2014; Plieger *et al*., 2014; Weiss *et al*., 2014; Jurczak *et al*., 2015; Koh *et al*., 2015, 2016; Kotyuk *et al*., 2015; Kruschwitz *et al*., 2015; Mezzavilla *et al*., 2015; Narita *et al*., 2015, 2018; Peciña *et al*., 2015; Roelofs *et al*., 2015; Voigt *et al*., 2015; Jung *et al*., 2016; Larsen *et al*., 2016; Madsen *et al*., 2016; Okbay *et al*., 2016; Petito *et al*., 2016; Powers *et al*., 2016; Schneider-Hassloff *et al*., 2016; Schneider-Matyka *et al*., 2016; Smith *et al*., 2016; Fan *et al*., 2017; Halldorsdottir *et al*., 2017; Iliadis *et al*., 2017; Lo *et al*., 2017; Viddal *et al*., 2017; Xu *et al*., 2017; Bîlc *et al*., 2018; Bizaoui *et al*., 2018; Chapman *et al*., 2018; Nagel *et al*., 2018, 2020; Noroña *et al*., 2018; Panitz *et al*., 2018; Sacchinelli *et al*., 2018; Terock *et al*., 2018, 2021; Turley *et al*., 2018; Yen *et al*., 2018; An *et al*., 2019; Chong *et al*., 2019; Luo *et al*., 2019; Michałowska-Sawczyn *et al*., 2019; Rodríguez-Ramos *et al*., 2019; Zou *et al*., 2019; Grzywacz *et al*., 2020; Hill *et al*., 2020; Hou *et al*., 2020; Kataja *et al*., 2020; Li *et al*., 2020; Morris *et al*., 2020; Salinas *et al*., 2020; Suchanecka *et al*., 2020; Tabak *et al*., 2020; Vaht *et al*., 2020; Zhao and Liu, 2020; Belonogova *et al*., 2021; Byrd *et al*., 2021; Heilbronner *et al*., 2021; Nestor *et al*., 2021) were finally selected: 27 GWAS and 175 observational genetic association studies. Of the latter, 142 were on neuroticism, 20 on alexitimia and 13 on emotional dysregulation (Fig. 1). Full results are shown in Supplementary Material S2, Supplemental Digital Content 1, http://links.lww.com/PG/A295, due to the unwieldy size of the result table. Below are the results concerning the most represented genes among the genetic association studies on neuroticism: serotonin transporter (SLC6A4, Table 1), catechol-ortho-methyltransferase (COMT, Table 2), monoamine oxidase type A (MAO-A, Table 3) and brain-derived neurotrophic factor (BDNF, Table 4). The outcomes of the screening on alexithymia are shown in Table 5 and on ED in Table 6. Results concerning GWAS studies are shown in Table 7.

**Table 1:** SLC6A4 review results (neuroticism)

Authors	Publication year	Tool used to assess neuroticism	Polymorphisms studied	Genetic association (S = short allele; L = long allele)	Sample population
Antypa and Van der Does	2010	NEO-PI-R	5-HTTLPR biallelic and triallelic	-	250 Dutch students (mostly females)
Ball *et al*.	1997	NEO-FFI	5-HTTLPR biallelic, STIN2-VNTR	-	2085 German twins
Brummett *et al*.	2003	NEO-PI-R	5-HTTLPR biallelic	L	103 depressed patients and 99 controls
Chang *et al*.	2017	MPI	5-HTTLPR triallelic (rs25531G coded equivalent to S)	S, both biallelic and triallelic (only in men)	1340 Taiwaneses
Chang *et al*.	2020	MPI	5-HTTLPR triallelic	S in males, L in females	2236 Han Chinese adults, including 736 patients with GAD and 1500 healthy participants
Deary *et al*.	1999	NEO-FFI	5-HTTLPR biallelic, STIN2-VNTR	-	809 men and 783 women from Scotland
Dragan and Oniszczenko	2006	NEO-FFI	5-HTTLPR biallelic	S	200 Polish (only females)
Du *et al*.	2000	NEO-FFI	5-HTTLPR biallelic	S (only in males)	186 Canadians
Flory *et al*.	1999	NEO-FFI	5-HTTLPR biallelic	-	271 Americans
Gelernter *et al*.	1998	NEO-FFI	5-HTTLPR biallelic, STIN2-VNTR	-	322 Americans
Greenberg *et al*.	2000	NEO-PI-R	5-HTTLPR biallelic	S	397 American sisters (thus females only)
Gustavsson *et al*.	1999	KSP	5-HTTLPR biallelic, STIN2-VNTR	-	Two healthy samples from Sweden (127 and 178)
Harro *et al*.	2009	EBBFI	5-HTTLPR biallelic	S	1176 Estonian children
Hettema *et al*.	2015	NEO-FFI, EPQ	5-HTTLPR biallelic, rs3813034, rs140701, rs6354, rs2020936	-	928 Americans
Jacob *et al*.	2004	NEO-PI, TPQ	5-HTTLPR biallelic	S (only within Cluster C patients)	320 patients with personality disorders (mainly cluster B and C) and 281 healthy controls, from Germany
Jang *et al*.	2001	NEO-PI-R	5-HTTLPR biallelic	S	388 American siblings
Jorm *et al*.	1998	EPQ-R	5-HTTLPR biallelic	-	759 Polish
Juhasz *et al*.	2010	NEO-PI-R, BFI	5-HTTLPR biallelic	-	1188 English (history of anxious or depressive psychopathology)
Jurczak *et al*.	2015	NEO-FFI	5-HTTLPR biallelic	-	272 healthy women from northern Poland in postmenopause
Kazantseva *et al*.	2008	EPI	5-HTTLPR biallelic, STIN2-VNTR	STIN2	301 healthy Russians
Kruschwitz *et al*.	2015	NEO-FFI	5-HTTLPR triallelic	S	178 Germans
Kuepper *et al*.	2012	FPI-R	5-HTTLPR triallelic	-	357 University students from Germany
Kuhnen *et al*.	2013	NEO-SF	5-HTTLPR biallelic	S	60 Americans
Lerman *et al*.	2000	EPI	5-HTTLPR biallelic	-	185 American smokers
Lovallo *et al*.	2014	EPQ	5-HTTLPR triallelic (rs25531G coded equivalent to S)	S (only in subjects with history of alchohol abuse disorder)	314 Americans
Luo *et al*.	2019	BFI	5-HTTLPR triallelic (rs25531G coded equivalent to S)	-	397 Americans
Madsen *et al*.	2016	NEO-PI-R	5-HTTLPR triallelic	-	76 healthy individuals from Denmark
Markus	2013	Dutch personality inventory	5-HTTLPR triallelic (rs25531G coded equivalent to S)	-	771 Dutch (mostly females)
Markus and Capello	2012	DPQ	5-HTTLPR triallelic	-	857 Dutch students
Middeldorp *et al*.	2007	ABV	5-HTTLPR biallelic	-	559 parents and 1245 twins, Dutch
Middeldorp *et al*.	2010	ABV	5-HTTLPR biallelic	-	126 fathers, 135 mothers, 87 monozygotic male twins, 161 monozygotic female twins, 238 DZ male twins/brothers and 408 DZ female twins/sisters from 438 families (Dutch)
Nestor *et al*.	2021	NEO-PI-R	5-HTTLPR biallelic	-	100 healthy participants from the USA
Pascual *et al*.	2007	ZKPQ	5-HTTLPR biallelic, STIN2-VNTR	-	65 borderline-disorder patients
Pelka-Wysiecka *et al*.	2012	NEO-FFI	5-HTTLPR biallelic	-	406 healthy Polish
Petito *et al*.	2016	NEO-FFI	5-HTTLPR biallelic	S	131 elite Italian athletes (only males)
Plieger *et al*.	2014	NEO-FFI	5-HTTLPR biallelic and triallelic	-	1075 Germans (mostly females)
Salinas *et al*.	2020	NEO-FFI	5-HTTLPR biallelic	S	76 patients from Chile with Borderline Personality Disorder (mostly females)
Samochowiec *et al*.	2004	NEO-FFI	5-HTTLPR biallelic	-	100 Polish
Schneider-Matyka *et al*.	2016	NEO-FFI	5-HTTLPR biallelic	-	214 women in postmenopause from northern Poland
Sen *et al*.	2004	NEO-PI	5-HTTLPR biallelic	S	384 Americans (mostly females)
Sher *et al*.	2000	NEO-PI-R	5-HTTLPR biallelic	S	236 Americans (mostly females)
Sirota *et al*.	1999	NEO-PI-R	5-HTTLPR biallelic	-	902 Americans
Stoltenberg *et al*.	2002	NEO-FFI	5-HTTLPR biallelic	-	161 individuals from families with history of alcoholism (USA)
Terracciano *et al*.	2009	NEO-PI-R	5-HTTLPR triallelic, rs1906451, rs435622, rs8076005, rs11080122, rs2020939, rs2020936, rs1487971, rs25603446	-	1182 Italians (Sardinia)
Umekage *et al*.	2003	NEO-PI-R	5-HTTLPR biallelic	-	244 Japanese (mostly females)
Verschoor *et al*.	2011	DPQ	5-HTTLPR triallelic	-	94 Dutch students selected from a larger sample for extreme values of neuroticism on both ends
Vinberg *et al*.	2010	EPQ	5-HTTLPR biallelic	S	204 high-risk and 204 low-risk twins (Danes)
Wachleski *et al*.	2008	MMPI	5-HTTLPR biallelic	-	67 patients with panic disorder from Brazil
Willis-Owen *et al*.	2005	EPQ	5-HTTLPR biallelic	-	1001 English (selected for extreme values of neuroticism on both ends)
Wray *et al*.	2009	EPQ, TPQ	rs2020934, rs6355, rs6354, rs2020936, 5-HTTLPR biallelic, rs28914832, rs140700, rs6355, rs6354, rs2020939, rs2020935, rs4251417, rs2020930, rs7214991, rs1050565, rs4251417	-	1161 depressed/anxious, 1051 controls, Australian twins

**Table 2:** Catechol-ortho-methyltransferase review results (neuroticism)

Authors	Publication year	Tool used to assess neuroticism	Polymorphisms studied	Genetic association	Sample population
Boscarino *et al*.	2012	NEO-FFI	rs4680	Not reported (sum of risk allele: -)	412 American chronic pain patients
Eley *et al*.	2003	NEO-FFI	rs4680	-	2085 German twins
Henderson *et al*.	2000	EPQ	rs4680	-	862 Australians and 1465 as replication sample
Hettema *et al*.	2015	NEO-FFI, EPQ-R	rs4680, rs165599	rs4680, rs4680-rs165599 (unreported allele, AA, females only)	928 Americans
Hoth *et al*.	2006	NEO-FFI	rs4680	-	486 healthy Americans
Kotyuk *et al*.	2015	NEO-FFI	rs4680	AA	616 elderly from the USA
Lehto *et al*.	2013	NEO-FFI	rs4680	GG (in 25 years old females; heterozygous were protected)	593 participants from Estonia split in three different age group: 15, 18, and 25 years
Luciano *et al*.	2010	NEO-FFI	rs4680	-	1641 elderly from Scotland
Olsson *et al*.	2005	NEO-FFI	rs4680	-	962 adolescents from Australia
Panitz *et al*.	2018	NEO-FFI	rs4680	-	383 Germans
Pelka-Wysiecka *et al*.		NEO-FFI	rs4680	GG (in males)	406 healthy Polish
Tochigi *et al*.	2006	NEO-PI-R	rs4680	Sum of risk allele	256 Japanese, employed in an hospital (mostly females)
Urata *et al*.	2007	NEO-FFI	rs4680	-	219 Japanese students (mostly females)
Wray *et al*.	2008	EPQ-R	rs4680	-	2045 Australians from 987 families

**Table 3:** Monoamine oxidase type A review results (neuroticism)

Authors	Publication year	Tool used to assess neuroticism	Polymorphisms studied	Genetic association	Sample population
Eley *et al*.	2003	NEO-FFI	MAO-A VNTR	>3.5 repetitions (in males)	2085 German twins
Jorm *et al*.	2000	EPQ-R	MAO-A VNTR	-	2725 Australians
Jurczak *et al*.	2015	NEO-FFI	MAO-A VNTR	-	272 healthy women from northern Poland in postmenopause
Pelka-Wysiecka *et al*.	2012	NEO-FFI	MAO-A VNTR	-	406 healthy Polish
Rodríguez-Ramos *et al*.	2019	BFI	MAO-A VNTR	>3 repetitions (only in females)	99 women from Spain
Samochowiec *et al*.	2004	NEO-FFI	MAO-A VNTR	-	100 Polish
Schneider-Matyka *et al*.	2016	NEO-FFI	MAO-A VNTR	-	214 women in post-menopause from northern Poland
Tochigi *et al*.	2006	NEO-PI-R	MAO-A VNTR	sum of risk alleles	256 Japanese, employed in an hospital (mostly females)
Urata *et al*.	2007	NEO-FFI	MAO-A VNTR, rs6323	-	219 Japanese students (mostly females)
Xu *et al*.	2017	MPI	rs3788862, rs5906957, rs979606	-	1160 men and 1180 women from the Netherlands

**Table 4:** Brain-derived neurotrophic factor review results (neuroticism)

Authors	Publication year	Tool used to assess neuroticism	Polymorphisms studied	Genetic association	Sample population
Gatt *et al*.	2007	NEO-FFI	rs6265	AA	169 controls and 39 subclinical depressed patients from Australia
Hünnerkopf *et al*.	2007	NEO-PI-R	rs6265	-	272 healthy volunteers from Germany
Joffe *et al*.	2009	NEO-FFI	rs6265	-	467 nonclinical Caucasian subjects
Jung *et al*.	2016	NEO-FFI	rs6265	AA in controls, GG in subjects who practiced meditation	64 controls and 72 subjects practicing meditation from Korea
Lang *et al*.	2005	NEO-FFI	rs6265	-	343 Germans
Lehto *et al*.	2016	EBBFI	rs6265	-	593 (age 15), 417 (age 18), 487 (age 25), all Estonian
Luciano *et al*.	2010	NEO-FFI	rs6265	-	1641 elderly from Scotland
Nestor *et al*.	2021	NEO-PI-R	rs6265	AA	100 healthy participants from the USA
Salinas *et al*.	2020	NEO-FFI	rs6265	-	76 patients from Chile with Borderline Personality Disorder (mostly females)
Sen *et al*.	2003	NEO-FFI	rs6265	GG	441 Americans
Tsai *et al*.	2004	TPQ	rs6265	-	114 healthy Chinese female volunteers
Willis-Owen *et al*.	2005	EPQ	rs6265	-	571 English people and 4843 Americans
Wray *et al*.	2008	EPQ-R	rs6265	-	2045 Australian twins sampled for extreme values of neuroticism on both ends from a larger sample

**Table 5:** Alexithymia review results

Authors	Publication year	Tool used to assess alexithymia	Genes	Polymorphisms studied	Genetic association	Sample population
Gong *et al*.	2014	TAS	5-HTR1A	rs6295	G	504 Chinese Han students
Ham *et al*.	2005	TAS	SLC6A4, COMT	rs4680, 5-HTTLPR biallelic	COMT: GG	109 students from Korea
Kano *et al*.	2012	TAS	SLC6A4	5-HTTLPR biallelic	L	304 healthy Japanese participants
Koh *et al*.	2016	TAS	COMT	rs4680	GG (both on total score and in subscores)	244 patients with OCD from Korea
Koh *et al*.	2015	TAS	OXTR	rs237885, rs237887, rs2268490, rs4686301, rs2254298, rs13316193, rs53576, and rs2268498	-	355 patients with obsessive-compulsive disorder (234 men, 121 women) from Korea
Li *et al*.	2020	TAS	5-HTR2A	rs6311	rs6311	602 Han Chinese
Mandelli and Serretti	2013	SPSS	SLC6A4	rs25531	-	64 Italians with alchohol and other substances abuse history
Roelofs *et al*.	2015	BVAQ	Rasopathies, Turner	Noonan Syndrome and Turner Syndrome (X0)	X0	Females only sample, Dutch. 40 individuals with Turner Syndrome, 40 with Noonan Syndrome and 40 controls.
Sacchinelli *et al*.	2018	TAS	SLC6A4	5-HTTLPR biallelic	-	115 healthy Italians
Schneider-Hassloff *et al*.	2016	CAS	OXTR	rs53576	-	195 Germans
Swart *et al*.	2011	BVAQ	COMT	rs4680	AA (only in verbalizing subscale)	493 undergraduate university students from Netherlands
Terock *et al*.	2018	TAS	SLC6A4	5-HTTLPR triallelic (rs25531G coded equivalent to S)	L	5283 Germans
Terock *et al*.	2021	TAS	VDBP	rs4588, rs7041	-	5783 Germans
Terock *et al*.	2021	TAS	5-HTR1A, 5-HTR2A	5HTR1A: rs6295 5-HTR2A: rs6311	-	3708 Germans
van Rijn *et al*	2006	BVAQ	Klinefelter	XXY	XXY	32 Klinefelter and 26 male controls (Dutch)
Voigt *et al*.	2015	TAS	BDNF, DRD2/ANKK1	rs1800497, rs6265	-	143 healthy individuals
Wahlstrom *et al*.	2012	TAS	DRD2/ANKK1	rs1800497	-	120 Undergraduate classified as ‘binge drinkers’ from the USA
Walter *et al*.	2011	TAS	BDNF, DRD2/ANKK1	rs6265, rs1800497	- (association present only in combination)	664 Germans
Wingbermühle *et al*.	2012	TAS, BVAQ	Rasopathies	Noonan Syndrome and Turner Syndrome (X0)	Noonan Syndrome	40 Dutch with Noonan Syndrome (various genes) and 40 controls
Zou *et al*.	2019	TAS	BDNF	rs6265	AA (both cases and controls)	223 Han Chinese people with panic disorder and 218 controls

**Table 6:** Emotion dysregulation review results

Authors	Publication year	Tool used to assess emotion dysregulation	Genes	Polymorphisms studied	Genetic association	Sample population
Amstadter *et al*.	2012	BIRD	SLC6A4, COMT	5-HTTLPR biallelic, rs4680	5-HTTLPR biallelic, rs4680 (distress tolerance)	218 early adolescents
Bîlc *et al*.	2018	Tasks	BDNF	rs6265	-	266 students from Romania
Byrd *et al*.	2020	PAI-BOR	OXTR	rs53576, rs2254298	rs53576 (GG)	2450 American female children (5-8 years)
Halldorsdottir *et al*.	2017	CERQ	FKBP5	rs9296158, rs3800373, rs1360780, rs9470080	-	1345 genotyped adolescents of Portuguese descent
Jorm *et al*.	2000	STSC	SLC6A4	5-HTTLPR biallelic	-	660 Australian children (3-15 years)
Kataja *et al*.	2020	Tasks	TPH2	rs4570625	-	330 Finnish children (8 months of age)
Kochanska *et al*.	2009	Tasks	SLC6A4	5-HTTLPR biallelic	- (association present only in ‘insecurely attachment’ sample)	89 American children
Murakami *et al*.	2009	Tasks	SLC6A4	5-HTTLPR biallelic	S	24 undegraduate and graduate students from Japan
Noroña *et al*.	2018	DCS	SLC6A4	5-HTTLPR triallelic (rs25531G coded equivalent to S)	-	99 children aged 3 from the USA
Propper *et al*.	2012	ERC (assessed by teachers)	DRD2, DRD4	DRD4: VNTR EX3, DRD2: rs1800497	rs1800497 (in males)	206 children, evaluated through ERC by teachers and parents.
Viddal *et al*.	2017	ERC	SLC6A4	5-HTTLPR biallelic	S (association present only at age 6)	602 Norwegian children
Weiss *et al*.	2014	SEAS	SLC6A4, COMT	5-HTTLPR biallelic COMT: rs4680	COMT: G (in the Intra-personal Domain)	289 healthy women from Germany
Yen *et al*.	2018	ASQ	ER	ESR α-Xbal	G	100 Taiwanese women with PMMD; 96 controls

**Table 7:** Genome-wide association studies review results

References	Author (s)	Publication year	Tool used to assess neuroticism	Highly significant results a	Summary of overall results concerning trait of interest^b^	Sample population	Trait
The Heidelberg Five’ personality dimensions: Genome-wide associations, polygenic risk for neuroticism, and psychopathology 20 years after assessment	Heilbronner *et al*.	2021	A combination of questionnaires including EPI, STAI-S and others defining a condition interpreted as ‘Emotional Lability’ (ELAB)	-	rs112852264, rs34464186, rs12746528, rs66583506, rs12750662 (unknown category) intergenic: rs2344174, rs34351096, rs17131127, rs35147867 intron: rs7001432 (CSMD1)	HeiDE study (‘Heidelberger Langzeitstudie zu Risikofaktoren und Diagnose chronischer Erkrankungen’); 5133 Germans	EMOTIONAL DYSREGULATION
A genome scan of neuroticism in nicotine dependent smokers	Neale *et al*.	2005	EPQ	-	-	129 families (843 siblings) from the USA	NEUROTICISM
A genome-wide association study of emotion dysregulation: Evidence for interleukin 2 receptor alpha	Powers *et al*.	2016	EDS	Intron: rs6602398 (ILR2A, only in males)	Intron: rs6602398 (ILR2A, only in males)	2600 African American from the USA	EMOTIONAL DYSREGULATION
A genome-wide association study of neuroticism in a population-based sample	Calboli *et al*.	2010	EPQ	-	6 genes: MGC57346, MSRA, XKR6, C17ORF69, KIAA1267 (integrative analysis of genomic and transcriptomic data of genome-wide association study (GWAS) and expression quantitative trait locus (eQTL) study)	UK Biobank, Psychiatric Genomics Consortium	NEUROTICISM
A genome-wide linkage study of individuals with high scores on NEO personality traits	Amin *et al*.	2012	NEO-FFi	-	-	2657 Dutch	NEUROTICISM
A genome-wide scan for Eysenckian personality dimensions in adolescent twin sibships: psychoticism, extraversion, neuroticism, and lie	Gillespie *et al*.	2008	EPI	-	Regions on chromosome 16 and 19	1280 Australian adolescent twins and their siblings	NEUROTICISM
A whole genome association study of neuroticism using DNA pooling	Shifman *et al*.	2008	EPQ-R	-	-	2000 English people and 1500 English people for replication sample: all of them oversampled for high neuroticism scores	NEUROTICISM
Analysis of functional variants reveals new candidate genes associated with alexithymia	Mezzavilla *et al*.	2015	TAS		Exons: rs144957058 (TMEM88B), rs45575636(ABCB4), rs35942033 (TP53AIP1), rs35287114 (ARHGAP32)	585 healthy adults from the Friuli Venezia Giulia Genetic Park project	ALEXITHYMIA
Association analysis in over 329,000 individuals identifies 116 independent variants influencing neuroticism	Luciano *et al*.	2018	EPQ-R-S	Intergenic: rs72694263, rs7107356, rs7111031, rs2953805, rs10097870, rs2921036 intronic: rs169235 (CACNA1E), rs1521732 (LINGO2, LOC105376004), rs1422192 (LINC00461), rs7175083 (LINGO1), rs7502590 (BAIAP2), rs11082011 (CELF4), rs6982308 (MSRA), rs7005884 (XKR6) UTR3: rs11090045 (ZC3H7B) UTR3: rs11090045 (ZC3H7B)	116 loci	UK Biobank	NEUROTICISM
Gene-based association analysis identifies 190 genes affecting neuroticism	Belonogova *et al*.	2021	EPQ-RS	57 noncoding variants; Exons: TRIM39-RPP21, RPP21, C12orf49	190 genes	UK Biobank	NEUROTICISM
Genetic contributions to two special factors of neuroticism are associated with affluence, higher intelligence, better health, and longer life	Hill *et al*.	2020	EPI	10 intron SNPs, 7 intergenic SNPs, 1 UTR3 (PAX6), 1 exon (DCAF5), 1 upstream (FBXL17)	51 loci	UK Biobank	NEUROTICISM
Genetic variants associated with subjective well-being, depressive symptoms, and neuroticism identified through genome-wide analyses	Okbay *et al*.	2016	EPI	Intron: rs10960103 (AKAP8P1,JKAMPP1), rs4938021 (LOC105369501), rs139237746 (SBF2), rs1557341 (CELF4), rs12938775 (PAFAH1B1), rs12961969 noncoding transcript variant: rs35688236 (LOC102724048), rs2150462, rs12903563, rs2572431 (inversion-tagging polymorphism on chromosome 8), rs193236081b (Inversion-tagging polymorphism on chromosome 17)	11 loci	UK Biobank, Psychiatric Genomics Consortium, Resource for Genetic Epidemiology Research on Aging	NEUROTICISM
Genome-wide analysis of over 106 000 individuals identifies 9 neuroticism-associated loci	Smith *et al*.	2016		Intergenic: rs490647, rs12637928, rs12378446, rs4977844 intron: rs4653663 (VRK2), rs62353264 (TMEM192), rs12682352 (MFHAS1), rs111433752 (LINC02210-CRHR1), rs1187264 (RPL12P40)	9 loci	UK Biobank, Scottish Family Health Study (6659), Queensland Institute of Medical Research Berghofer Medical Research Institute cohort (8687)	NEUROTICISM
Genome-wide association analysis followed by a replication study implicates a novel candidate gene for neuroticism	van den Oord *et al*.	2008	EPQ-R	-	-	1227 people from the USA	NEUROTICISM
Genome-wide association scan for five major dimensions of personality	Terracciano *et al*.	2010	NEO-PI-R	-	rs3026815, rs6047641, rs1159275, rs7329003	6148 people from an isolated community in Sardinia, 3900 in the replication sample	NEUROTICISM
Genome-wide association study of the five-factor model of personality in young Korean women	Kim *et al*.	2013	NEO-PI-R	-	GWAS: OR1A2	1089 Korean women	NEUROTICISM
Genome-wide association study of the sensitivity to environmental stress and adversity neuroticism cluster	Nagel *et al*.	2020	EPQ	-	47 loci	UK Biobank	NEUROTICISM
Genome-wide association uncovers shared genetic effects among personality traits and mood states	Luciano *et al*.	2012	EPQ	-	Genes: LCE3C, SCAMP2, POLR3A, ULK3, LMAN1L	Croatia (800), Scotland (400), England (1500), Netherlands (1300)	NEUROTICISM
Integrating genome-wide association study and expression quantitative trait loci data identifies multiple genes and gene set associated with neuroticism	Fan *et al*.	2017	EPI	-		UK Biobank, Psychiatric Genomics Consortium	NEUROTICISM
Integrative analysis of genome-wide association study and common meQTLs for exploring the effects of DNA methylation on the development of neuroticism	Zhao and Liu	2020	EPI	-	11 genes identified through integrative analysis	UK Biobank, Psychiatric Genomics Consortium	NEUROTICISM
Item-level analyses reveal genetic heterogeneity in neuroticism	Nagel *et al*.	2018	EPQ-R-SF	117 loci (not reported any information on subdivision between introns, intergenic or exons)	117 loci associated with overall neuroticism score, 138 item-specific	UK Biobank	NEUROTICISM
Linkage analysis of extremely discordant and concordant sibling pairs identifies quantitative-trait loci that influence variation in the human personality trait neuroticism	Fullerton *et al*.	2003	EPQ	-	Five regions: 1q, 4q, 7p, 12q and 13q	34580 sibling pairs in the southwest of England	NEUROTICISM
Modeling prior information of common genetic variants improves gene discovery for neuroticism	Lo *et al*.	2017	BFI	Intergenic: rs12102100 intron: rs9822731 (CADM2), rs17022974 (CADM2), rs10812851 (LINGO2, LOC105376004), rs9611505 (EP300)	Combination of relative enrichment score (RES) and conditional false discovery rate (FDR): CADM2, LINGO2 and EP300	23 and Me database, UK Biobank, Psychiatric Genomics Consortium	NEUROTICISM
Multitrait analysis of genome-wide association summary statistics using MTAG	Turley *et al*.	2018	EPI	Noncoding transcript variant rs2572431(LINC00529), rs1187229 (LOC105372072, LOC105372073) intron: rs62057143 (CRHR1, LINC02210-CRHR1), rs10960103 (AKAP8P1,JKAMPP1), rs60827133, rs4938021 (LOC105369501), rs1557341 (CELF4), rs8084351 (DCC), rs10733389, rs10113343 (PINX1), rs9893575 (LOC105371490), rs716804 (SBF2)	37 loci	UK Biobank	NEUROTICISM
Pathway analysis of genome-wide association datasets of personality traits	Kim *et al*.	2015	NEO-PI-R	L1CAM pathway and associated genes	Pathway analysis: L1CAM pathway and associated genes	1089 Korean women	NEUROTICISM
The influence of X chromosome variants on trait neuroticism	Luciano *et al*.	2021	EPQ-R-SF	intergenic: rs6630665, rs764018176, rs177010 intronic: rs5977754 (HS6ST2)	204 loci on chromosome X	UK Biobank	NEUROTICISM
TMPRSS9 and GRIN2B are associated with neuroticism: a genome-wide association study in a European sample	Aragam *et al*.	2013	NEO-FFI	-	GWAS: TMPRSS9	NESDA cohort 2008 (2000 Dutch)	NEUROTICISM

a *P* equal or lower than 10^−8^.

b If not otherwise specified, *P* value threshold: 5 × 10^−6^.

For each category, the following number of studies was found: SLC6A4 (50), COMT (13), MAO-A (9) and BDNF (12). The average risk of bias score was equal to 20.13 (SD: 4.2).

The most commonly used questionnaires for neuroticism were the NEO-PI and the Eysenck Personality Inventory (Questionnaire). Alexithymia in our sample was assessed more commonly through the TAS-20 and the BVAQ. Genetic association studies evaluating emotional dysregulation used a wider variety of different tools (see Table 6); further considerations on this trait should take into account this variability.

Since GWAS results are too vast to be meaningfully discussed, data with *P* ≤ 10^−8^ was given special consideration. This threshold is considered the standard in newer GWAS, despite suggestions to lower it to 10^−7^ (Chen *et al*., 2021).

Table 8 contains the questionnaire abbreviations. Risk of bias scores are shown in Supplementary Material S1, Supplemental Digital Content 2, http://links.lww.com/PG/A296.

**Table 8:** Questionnaires abbreviations

Abbreviation	Trait	Questionnaire
NEO-PI-R	Neuroticism	NEO Personality Inventory-Revised
ABV	Neuroticism	Amsterdamse Biografische Vragenslijst
BFI	Neuroticism	Big Five Inventory
EBBFI	Neuroticism	Estonian Brief Big Five Inventory
NEO-FFI	Neuroticism	NEO Five-Factor Inventory
MPI	Neuroticism	Maudsley Personality Inventory
EPQ	Neuroticism	Eysenck Personality Questionnaire
EPQ-R	Neuroticism	Eysenck Personality Questionnaire-Revised
DPQ	Neuroticism	Dutch Personality Questionnaire
ZKPQ	Neuroticism	Zuckerman-Kuhlman Personality Questionnaire
EPI	Neuroticism	Eysenck Personality Inventory
TPQ	Neuroticism	Temperament Personality Questionnaire
FPI-R	Neuroticism	Freiburg Personality Inventory-Revised
KSP	Neuroticism	Karolinska Scales of Personality
NEO-SF	Neuroticism	NEO Short Form
NEO-PI	Neuroticism	NEO Personality Inventory
TAS	Alexithymia	Toronto Alexithymia Scale
BVAQ	Alexithymia	Bermond–Vorst Alexithymia Questionnaire
SPSS	Alexithymia	Schalling-Sifneos Personality Scale
CAS	Alexithymia	Childhood Attachment Security
ERC	Emotional dysregulation	Emotion Regulation Checklist
STSC	Emotional dysregulation	Short Temperament Scale for Child
SEAS	Emotional dysregulation	Self-report Emotional Ability Scale
DCS	Emotional dysregulation	Dysregulation Coding System
STAI-S	Emotional dysregulation	State form of the State-Trait Anxiety Inventory
ASQ	Emotional dysregulation	Affective Style Questionnaire
CERQ	Emotional dysregulation	Cognitive Emotion Regulation Questionnaire
PAI-BOR	Emotional dysregulation	Personality Assessment Inventory-Borderline Scale
BIRD	Emotional dysregulation	Behavioral Indicator of Resilience to Distress
EDS	Emotional dysregulation	Emotion Dysregulation Scale

## Discussion

In order to treat the topics in a hierarchical fashion, we decided to prioritize describing results concerning genes that were studied in 10 or more genetic association analyses in their own category. In the successive sections, we proceeded to explore the associations between neuroticism and less studied genes, and finally to discuss the possible genetic basis of the remaining traits.

### Neuroticism: SLC6A4, COMT, MAO-A and BDNF

According to the results of our systematic review, no solid association seemed to emerge between neuroticism and these very thoroughly studied genes (Mandelli and Serretti, 2013). These genes have understandably been at the epicenter of scientific attention since the beginning of genetic research in psychiatry (Collier *et al*., 1996; Eley *et al*., 2003; Strauss *et al*., 2004) because of their hypothetical and plausible importance in brain function, especially regarding cortical networks (Matsumoto *et al*., 2003; Martinowich *et al*., 2007; Alia-Klein *et al*., 2008), since their respective proteins are involved in basic neurotransmission and, most importantly, in what was currently believed to be the main mechanism of action of many drugs such as antidepressants and antipsychotics (Sundaram and Mahajan, 1980; Meltzer, 1991).

Despite no meta-analysis being carried out, it is possible to state that these genes might not have a strong connection with neuroticism. Most of the papers found no association; however, in some studies, despite no direct association, these genes were found to significantly increase neuroticism scores when combined with several moderating factors such as sex, sociodemographic status and other secondary factors such as cluster C personality disorder diagnosis (Jacob *et al*., 2004), meditation practice (Jung *et al*., 2016) and alcohol abuse (Lovallo *et al*., 2014). In some cases (Brummett *et al*., 2003; Sen *et al*., 2004; Kotyuk *et al*., 2015; Chang *et al*., 2017), conflicting evidence emerged regarding which of the two alleles is involved in increasing risk of high neuroticism scores, further weakening the potential connection between these alleles and neuroticism.

As will be more thoroughly discussed further on, no GWAS found any link between these four genes and neuroticism, further weakening their potential in moderating neuroticism.

### Neuroticism: other genes and genome-wide association study

Overall, more than 60 other genes were studied, most of them in single studies. Thirty-six of them were significantly associated with neuroticism (a full list of these genes can be found in Supplementary Materials, Supplemental Digital Content 1, http://links.lww.com/PG/A295). Some of these genes are conventionally connected in some fashion to brain function, such as the 5-HTR1A and 2A genes and DRD2, DRD3, DRD4 (Missale *et al*., 1998; Pytliak *et al*., 2011); others are transcription factors, adherence proteins or do not have a known function in the nervous system.

GWASs point towards a somewhat similar direction. Most of the studies found associations with intronic or even intergenic noncoding regions (see Supplementary Materials, Supplemental Digital Content 1, http://links.lww.com/PG/A295). This was to be expected, given that the majority of DNA sequences are represented by such regions (Francis and Wörheide, 2017); moreover, such regions are hypothesized to have a regulatory function on translated sequences. This can happen through several different mechanisms. There is growing evidence that intergenic regions might regulate chromatin remodeling (Mayer *et al*., 2006; Romanowska and Joshi, 2019) and act as enhancers (Ransohoff *et al*., 2018), thus allowing fine-tuning of more nuanced transcriptional equilibriums as compared to exons.

Most of the GWAS acquired some of their data from the same original dataset, the UK Biobank; as the Biobank was updated, though, the sample increased constantly in numbers. Moreover, several studies employed very interesting nonstandard statistical techniques, which show great promise. Multitrait analysis of GWAS (a variation of standard GWAS in which data on several traits is analyzed jointly in order to maximize detection rate), methylation analysis and pathway analysis are all helpful tools in order to increase both the precision and the scope of GWAS results.

Through pathway analysis, L1CAM, an intercellular signaling adhesion protein, was found to be implicated in increasing neuroticism (Kim *et al*., 2015). DCAF5 (Hill *et al*., 2020), coding for a protein involved in ubiquitin function regulation, is also linked to neuroticism scores. In the same study (Kim *et al*., 2015), another interesting association was PAX6, an embrional transcription factor. It is worth noting that, similarly to what was argued in the above paragraph, exons with regulatory functions on other sequences or biochemical mechanisms tend to be the most common findings when searching for gene-environment associations in the field of psychiatry. As further proof to this statement, polymorphisms in RBFOX1, a splicing-regulating protein, have been replicated in multiple GWAS (Okbay *et al*., 2016; Luciano *et al*., 2018; Nagel *et al*., 2018, 2020); variations in LINC00461, a noncoding RNA involved in miRNA and siRNA regulations, are as well a replicated finding (Okbay *et al*., 2016; Luciano *et al*., 2018; Nagel *et al*., 2018; Turley *et al*., 2018; Hill *et al*., 2020).

An association was found with two distinct chromosomal inversions (respectively on chromosomes 8 and 17) (Okbay *et al*., 2016). The exact mechanism by which these chromosomal alterations affect neuroticism is not clear. Widespread regulatory mechanisms disruption and alteration in 3D chromatin structure are a possible hypothesis; Okbay *et al*. (2016) suggest that the inversion might relocate some crucial regulatory regions.

A solid association can be assumed for CRHR1, since it was found in two GWAS and several genetic association studies. CRHR1 is the gene coding for the corticotropin-releasing factor receptor. As such, it is a key component of stress reaction and cortisol homeostasis. This receptor is also present in the brain and has been connected to satiety feelings (Tu *et al*., 2007) and depressive and anxiety symptoms (Rogers *et al*., 2013). No data could be found in literature regarding the exact effect of the variants linked to neuroticism; assuming that these variants decrease the efficacy of CRHR1 receptor, we can hypothesize that this mutation might lead to a reduction of hypothalamic control on cortisol production. A more direct mechanism can also be mentioned; several CRHR1 polymorphisms (not the one found in this review, though) were associated with cortisol reactivity in children (Sheikh *et al*., 2013).

Several other genes were identified in multiple GWAS. Among these, many have important roles in regulating the neural progenitors migration, such as deleted in colorectal cancer (DCC)(Okbay *et al*., 2016; Nagel *et al*., 2018; Turley *et al*., 2018; Hill *et al*., 2020), XKR6 (Okbay *et al*., 2016; Luciano *et al*., 2018; Hill *et al*., 2020) and transcription factor 4 (TCF4) (Okbay *et al*., 2016; Luciano *et al*., 2018; Nagel *et al*., 2018; Hill *et al*., 2020). DCC has also been established as a gene involved in increased impulsivity in children (Jiang *et al*., 2015) as well as colon cancer risk (Berke, 2018). Other genes have a less clear link to psychopathology, such as MSRA, coding for a protein involved in free-radicals metabolism (Okbay *et al*., 2016; Luciano *et al*., 2018; Nagel *et al*., 2018).

One of the most interesting gene is DRD2; despite mixed results among candidate-gene association studies, with positive association (Wacker *et al*., 2005; Jutras-Aswad *et al*., 2012; Grzywacz *et al*., 2020; Suchanecka *et al*., 2020) outweighing the null hypothesis (Hibino *et al*., 2006; Pełka-Wysiecka *et al*., 2012), GWAS studies replicated several times the finding of its association with neuroticism (Okbay *et al*., 2016; Luciano *et al*., 2018; Nagel *et al*., 2018, 2020; Turley *et al*., 2018; Hill *et al*., 2020). As mentioned previously, the DRD2 gene codes for a crucial dopamine receptor, currently regarded as being one of the main target of antipsychotics medications.

### Alexithymia

Data on alexithymia are sparse and less comprehensive compared with neuroticism. Thus, it is not possible to extract conclusive results on the subject. On the other hand, the assessment of alexithymia is extremely homogeneous, as nearly all included studies employed the TAS-20 questionnaire. Some relevant results can nevertheless be extracted.

Interestingly, SLC6A4 association with alexithymia emerged in two studies (Kano *et al*., 2012; Terock *et al*., 2018). Moreover, the risk factor was the short variant, which is commonly thought as having an influence on psychopathology, especially in the depression and anxiety spheres (Juhasz *et al*., 2015). Thus, more data on this peculiar gene-trait association are warranted and needed. Though, it must be underlined that three other studies found no association between SLC6A4 and alexithymia.

Other intriguing associations are those with some chromosomal abnormalities such as Turner and Klinefelter syndromes. Increased psychiatric burden is not a novelty in these syndromes.

For example, there is evidence of higher levels of alexithymia in Turner syndrome if compared with Noonan syndrome and healthy controls (Roelofs *et al*., 2015).

Other studies concerning Klinefelter syndrome pointed out higher levels of psychological distress, such as depression, paranoid ideation, phobias, psychoticism and obsessive thoughts, and a central role of alexithymia in the development of these aspects (Skakkebæk *et al*., 2018; Giagulli *et al*., 2019; van Rijn and Swaab, 2020; Fabrazzo *et al*., 2021). It is well known in current literature that Klinefelter syndrome might also predispose to the development of maladaptive psychological constructs, such as obsessive-compulsive symptoms associated with lower total, verbal and performance IQ scores, although there is no clear evidence on etiopathological process and if these symptoms are due to genetic makeup or environment and social stigma (Fisher *et al*., 2015).

These syndromes are known to be associated with a variable degree of social functioning impairment (van Rijn *et al*., 2018); it is, therefore, worth considering that the areas of performance more commonly affected might predispose to a specific personality trait (with the genetic footprint as a unifying mark).

Noonan syndrome is a cluster of monogenic conditions involving several genes in the RAS-MAPK pathway (PTPN11, SOS1, SHOC2, MAP2K1/2 and KRAS in the included paper).

Also, patients with Noonan syndrome showed higher levels of introversion, alexithymia, anxiety and depression, which may predispose to internalizing problems (Roelofs *et al*., 2015; Roelofs *et al*., 2020).

There is also evidence that global and social functioning is negatively correlated with family quality of life and a negative environment (neglect) (Davico *et al*., 2022), pointing out that environmental factors, in this case, play a relevant role.

A single GWAS was found studying alexithymia (Mezzavilla *et al*., 2015). Interestingly, this study found several exonic associations: TMEM88B (a transmembrane protein, most likely involved in intercellular signaling), ABCB4 [a transporter protein that is linked to some forms of cancer and multidrug resistances (Nayagam *et al*., 2020, p. 1)], TP53AIP1 (whose protein is part of the p53 pathway) and ARHGAP32 from the Rho G protein pathway. All of these proteins have very indirect known effects on synapses; two of them, namely, ABCB4 and TP53AIP1, might be involved in neurogenesis of the Novo neurons or in organizing cortical architecture.

### Emotion dysregulation

ED assessment in the included studies was far from homogenous (see Table 6 and Introduction section). Evidence drawn from this review must, therefore, be carefully evaluated taking into consideration this fact.

SLC6A4 was the most represented among genes investigated regarding ED. Some theoretical models were proposed in 2007 (Canli and Lesch, 2007) trying to link neuroscience evidence with the genetic data available at the time. The main idea was that SLC6A4 functional variants might induce the amygdala nuclei to be more or less reactive to external stimuli, thus inducing a stronger or weaker emotional response according to the polymorphism. Since our review did not focus on endophenotypes but rather on a more direct association with the trait of interest, we can not undermine or prove such hypothesis. Seven gene-association studies were found regarding SLC6A4 and its relation with ED (mostly in children and adolescents, Table 6); four supported the null hypothesis (Jorm *et al*., 1998; Kochanska *et al*., 2009; Weiss *et al*., 2014; Noroña *et al*., 2018), whereas two reported an association with the short allele and one reported an association with a polymorphism (rs4680) (Murakami *et al*., 2009; Amstadter *et al*., 2012; Viddal *et al*., 2017). Moreover, GWAS studies on ED did not report association with SLC6A4 (more details in later paragraphs).

Two studies found associations with COMT (only in specific subscales) (Weiss *et al*., 2014; Amstadter *et al*., 2012).

An association with the oxytocin receptor was found among a large sample of female children (Byrd *et al*., 2021). In fact, it is not unreasonable to assume that such a gene might have psychological and psychiatric implications. Interestingly, oxytocin blood and brain level seem to be connected to both prosocial behavior and anger reactions (Aragón *et al*., 2015; Byrd *et al*., 2021) and, most importantly in this context, to empathy (Barchi-Ferreira and Osório, 2021).

Our search provided two GWAS eligible for discussion on ED (see Table 7). Both have relatively small samples. One of them (Powers *et al*., 2016) found a single association with an intronic regulatory region of ILR2A, the gene coding for the interleukin 2A receptor. There is also other evidence linking this receptor to psychiatric conditions in general (Nässberger and Träskman-Bendz, 1993; Rapaport and Stein, 1994) and even directly to alexithymia (Gil *et al*., 2007; De Berardis *et al*., 2014). A plausible explanation as to why an interleukin gene could potentially have an impact on personality can be hypothesized through microglia activity in synaptic pruning and modulation in general. The importance of these often neglected cells in brain circuitry homeostasis has already been proven in several neurologic conditions such as Alzheimer's and multiple sclerosis (Augusto-Oliveira *et al*., 2019; Li *et al*., 2022) and even in psychotic disorders (Germann *et al*., 2021). It is, therefore, not unreasonable to assume that interleukin 2A might influence microglia actions on synaptic remodeling and thus affect personality.

### Limitations

Several factors must be accounted for when proposing an interpretation of our results. The diverse nature of assessment tools increases potential biases; a more standard approach in order to measure under-investigated traits such as ED is warranted. An example of such an attitude can be seen in alexithymia research, with the TAS-20 widely being recognized as the main questionnaire.

Another limitation is that most of the studies reviewed did not explain how the study size was arrived at: the reason for this lack of conformity probably lies in the fact that the aforementioned studies did not include a power analysis in their design. Furthermore, characteristics of study participants, such as demographics and social information, were not provided in a majority of the selected studies.

### Conclusion

DNA is mostly made of such apparently ‘junk’ regions; as research in genomics and proteomics advances, though, more and more evidences are mounting up to prove the crucial role that intronic and intergenic regions actually have in transcriptional regulation, splicing, chromatin density regulation, methylation and finer modulation mechanisms.

In fact, it could even be hypothesized that the ‘major’ receptor genes are too widespread among the brain, and thus, a mutation in such genes might prove to be too constraining to effectively influence personality and other more subtle psychological traits. On the other hand, a neuroscientific model taking into account millions of base pairs scattered across all chromosomes and constantly influencing both exon and each other’s activity might constitute a better framework and foundation to better understand the way the human brain works. This point of view also has a stronger phylogenetic basis as compared with a model that only encompasses few loci; in fact, having a larger portion of DNA devoted to fine regulation and modulation of brain function allows evolutionary mechanisms more room to act and apply selective pressure (be it positive or negative) that would in turn influence the architecture of a complex organ such as a brain.

This theoretical approach is supported by GWAS results in general, and by GWAS results in neuroticism especially. As detailed in the Discussion section, even when accounting only exonic mutations, most of these affect regulatory and transcriptional proteins (i.e. LINC00461, RBFOX1 and L1CAM) rather than, for example, ion channels or receptors. Evaluating the biochemical impact of such proteins is a complex endeavor that GWAS studies alone cannot fully address; nevertheless, such studies can inform the direction of protein-functionality studies.

Key neurodevelopmental proteins are also potentially involved in the neuroticism pathogenesis, as pointed out by polymorphisms in genes such as DCC, XKR6 and TCF4. Scientific literature, in fact, supports the hypothesis that neuroticism’s phenotype might develop quite early in life (Zupančič and Kavčič, 2013; Ask *et al*., 2021). Brain structure differences in high neuroticism children, namely in several whole-brain parameters as well as the prefrontal, occipital and lateral temporal cortex (Restrepo-Lozano *et al*., 2022) have also been reported, despite polygenic risk score associations being inconclusive.

Even though the majority of findings regarded nonconding regions or regulatory exons, GWAS on neuroticism identified significantly and replicated polymorphisms in two key brain receptors: CRHR1 and DRD2. These findings provide an interesting direction for future research, as these receptors’ function is relatively well-known in the brain (Rogers *et al*., 2013; Berke, 2018).

Little GWAS data are currently available on alexithymia and emotional dysregulation; more research is needed in this area, as the data currently available already yielded some interesting results (TMEM88B, ABCB4 and TP53AIP1 for alexithymia, and ILR2A for emotional dysregulation). Since the studies had small samples for GWAS standards (Hong and Park, 2012; Haram *et al*., 2014; Powers *et al*., 2016; Heilbronner *et al*., 2021) it is likely that more associations could be detected in the future.

Neuroticism, alexithymia and emotional dysregulation constitute key concepts both in psychology research and clinical practice among psychiatrists. These traits influence treatment outcome and prognosis from a variety of directions: therapeutic alliance, compliance with medication and social functioning. Being this the case, understanding their etiopathology and genesis is of crucial importance. Clearly, as is often the case in psychiatry and brain research, genetic factors cannot fully explain the variability of the phenomena by themselves; the environment must play a key role as well. Nevertheless, a full understanding of the genetic side of the coin is necessary and might have huge clinical implications, both directly and indirectly. Genetic panels predicting psychopathological risk might be developed, and pharmaceutical research can and will benefit greatly from a deeper knowledge of these genetic mechanisms. Such mechanisms can be unveiled through additional research, especially in the form of GWAS.

## Acknowledgements

G.P.M. conceived the study, with the coordination offered by G.C., E.C and E. R. for the study design. G.P.M. designed the search algorithm, with the support of. E.C., O.B.B. and V.P.; G.P.M., O.B.B, B.B. and V.P. collected the data and performed the screening process. All authors contributed to the interpretation of the studies and to the synthesis of results. The first draft was written by G.P.M, V.P., B.B. and O.B.B. under the supervision of G.C., V.B., S.S., B.N. and V.R.; the final manuscript was approved by all the authors.

Protocol and registration: methods of the analysis and inclusion criteria were specified in advance and documented in a protocol. The protocol, published in advance, can be retrieved as Prospero ID CRD42021267732.

Availability of data, code and other materials: the database of the studies, with the extracted data items, can be shared upon reasonable request to the corresponding author.

### Conflicts of interest

There are no conflicts of interest.

## Supplementary Material




