# Supplementary figures and images for: Emotional dysregulation, alexithymia and neuroticism: a systematic review on the genetic basis of a subset of psychological traits
Source: Psychiatr Genet. 2022 Dec 20;33(3):79–101. doi: 10.1097/YPG.0000000000000335 (PMC10158611; doi:10.1097/YPG.0000000000000335)

## S1: Risk of bias

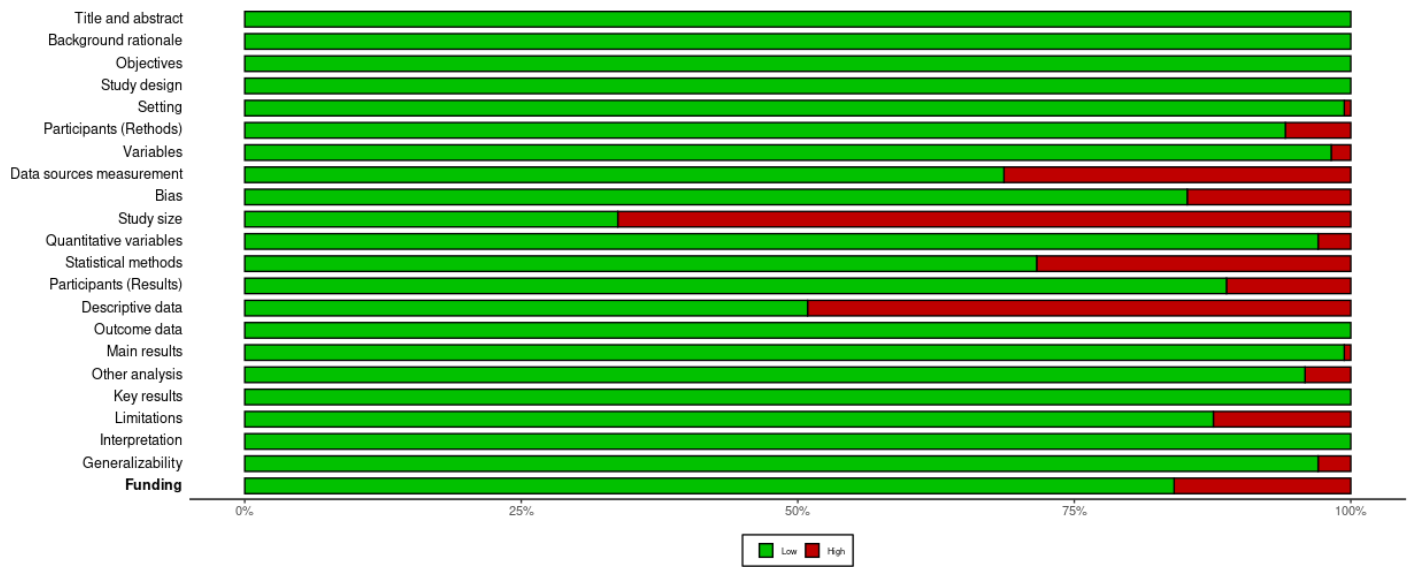

Supplement: Supplementary file 2 [file pg-33-79-s002.pdf]
